# Changes in Mitochondrial Membrane Potential in *In Vitro* Electroporation with Nano- and Microsecond Pulses

**DOI:** 10.1089/bioe.2024.0007

**Published:** 2024-06-12

**Authors:** Tamara Polajžer, Wencheng Peng, Chenguo Yao, Damijan Miklavčič

**Affiliations:** ^1^ Faculty of Electrical Engineering, University of Ljubljana, Ljubljana, Slovenia.; ^2^ The State Key Laboratory of Power Transmission Equipment and System Security and New Technology, School of Electrical Engineering, Chongqing University, Chongqing, Republic of China.

**Keywords:** electroporation, nanosecond pulses, microsecond pulses, mitochondria, mitochondrial membrane potential

## Abstract

With the introduction of nanosecond (ns) pulses, it was suggested that such pulses could be used to permeabilize intracellular membranes, including the mitochondrial membrane. The results presented thus far, however, are not conclusive. Interestingly, the effect of longer microsecond (μs) pulses on changes in mitochondria has never been investigated. We, therefore, investigated the changes in mitochondrial membrane permeability through changes in mitochondrial membrane potential (MMP) in CHO and H9c2 cells after electroporation with 4 ns, 200 ns, and 100 μs pulses. In the range of reversible electroporation, the decrease in MMP generally depended on the cell line. In CHO, ns pulses decreased MMP at lower electroporation intensities than μs. In H9c2, ns and μs were equally effective. In the range of irreversible electroporation, MMP decreased even further, regardless of pulse duration and cell type. The analysis at different time points showed that the changes in MMP within the first hour after pulse treatment are dynamic. Our results on the efficacy of ns pulses are consistent with published data, but with this study we show that μs pulses cause similar changes in MMP as ns pulses, demonstrating that electroporation affects MMP regardless of pulse duration. At the same time, however, differences in MMP changes were observed between different cell lines, indicating some dependence of MMP changes on cell type.

## Introduction

Application of electroporation pulses results in structural and chemical changes in the plasma membrane, which leads to increase in plasma membrane permeability.^1^ For a long time, the pulse duration was limited to milliseconds (ms) and microseconds (μs) at low voltage due to technical limitations. In 2000s it was first suggested that nanosecond (ns) pulses could be used to permeabilize intracellular membranes without affecting plasma membrane integrity.^2^ This was followed by experimental studies, showing changes in membrane permeability of intracellular organelles in the absence of changes in plasma membrane permeability,^2,3^ although it was later shown theoretically and experimentally that also plasma membrane is affected.^4–17^ Nonetheless, ns electroporation has become of interest in electroporation due to permeabilization specifics, focusing mostly on mitochondria, as mitochondria are involved in multiple signaling pathways and are crucial for ATP production.^18^

The mitochondria consist of two membranes—inner, which is extensively folded into cristae, and outer membrane. Between inner and outer membranes is an intermembrane space, where apoptogenic molecules are present and separated from the cytosol.^19^ Generally, in regulated cell death, mitochondrial outer membrane permeabilization (MOMP) occurs by forming pores in outer mitochondrial membrane and releasing apoptotic protein into cytosol, triggering cell death.^20^ This can be either apoptosis or necrosis, depending on ATP generation capacity of the cell.^21^ In electroporation with ns pulses, the reason for pore formation could be different, but with the same result—a release of apoptotic proteins. The release of cytochrome C and other apoptotic markers after ns electroporation treatment has been shown before.^3,4,13,22,23^

Mitochondria produce ATP. The mitochondrial membrane potential (MMP, ΔΨm) generated by proton pumps is an essential component in the process of ATP synthesis during oxidative phosphorylation.^24^ If MMP decreases, the flow of H^+^ across the inner mitochondrial membrane is impaired, followed by the reduction of driving force needed for ATP synthesis.^25^ In response to ATP depletion, calcium overloads, which may lead to cell death.^26^ Changes to mitochondrial membrane permeability can be observed through changes in mitochondrial membrane potential (MMP). MMP is commonly measured by fluorescent dyes, which allows monitoring its changes.^27^ Changes in MMP after electroporation with ns pulses have been investigated using different fluorescent dyes [TMRE, JC-1, Rhodamine 123, Mitotrackers, Dihexyloxacarbocyanine Iodide (DiOC_6_(3))].^4,22,28–38^ Even though analysis of MMP was performed with different MMP dyes at different time points (from minutes to hours) after pulse treatment, they all show the same trend: after ns pulse treatment MMP decreases with the increase of electroporation intensity. Furthermore, in ns pulse studies also involving viability experiments decreased MMP was observed above the threshold of irreversible electroporation.^22,31^ However, there is no conclusive evidence available demonstrating that decreased MMP results from direct ns electroporation of inner mitochondrial membrane. It is possible also that molecular influx due to plasma membrane permeabilization could lead to MMP changes or could transition as a secondary effect following the plasma membrane permeabilization by electroporation.^4,39^

In a recent study, we showed that cells respond to ns pulse treatment similarly to other pulse treatments (ms, μs, high-frequency irreversible electroporation [HFIRE]) with respect to cell death dynamics.^40,41^ Nanosecond pulses were also shown to be comparably effective as longer pulses in gene transfer^42^ and in electrochemotherapy.^43^ If cells respond to nano and longer pulses similarly with respect to cell death, if both nano and longer pulses can be used for electroporation therapies, and if ns pulses can affect organelle and plasma membrane, how sure are we that longer pulses cannot affect both organelle and plasma membrane as well? Interestingly the effect of longer pulses on changes in mitochondria was never investigated. To the best of our knowledge, this is the first study investigating changes in mitochondrial membrane permeability assessed through changes in MMP comparing ns and μs pulses.

## Materials and Methods

Chinese hamster ovary cells (CHO) and rat heart myoblast cells (H9c2) were purchased from European Collection of Authenticated Cell Cultures. CHO cells were grown in HAM F-12 growth medium (PAA, Austria) and H9c2 in Dulbecco’s modified Eagle’s medium (DMEM) growth medium (Sigma-Aldrich, USA). Both media were supplemented with 10% fetal bovine serum (FBS, Sigma-Aldrich, USA), l-glutamine (0.5% for CHO, 2% for H9c2) (StemCell, Canada), penicillin/streptomycin (PAA, Austria), and 0.1% gentamicin (Sigma-Aldrich, USA). Cells were grown in an incubator at 37°C with controlled atmosphere (CHO at 5% CO_2_, H9c2 at 10% CO_2_) until 70–80% confluency was reached. For experiments, growth medium was removed and the trypsin-EDTA (PAA, Austria) was added to detach cells. After 3 min, fresh medium was added to inactivate trypsin. Cell suspension was then centrifuged at 180 *g* for 5 min, supernatant was removed, cells were resuspended in fresh growth medium to a desired density (1 × 10^6^ cells/mL for 200 ns and 100 μs pulse treatment; 3 × 10^6^ cells/mL for 4 ns pulse treatment). Due to impedance matching of 4 ns pulse generator, sample volume was limited to 60 μL. Therefore, to have approximately the same number of exposed cells in each treatment, cell density for 4 ns was increased three times. This is still a dispersed cell solution where we do not expect that the induced transmembrane voltage would be reduced due to neighboring effect; this occurs at densities higher than 10^8^ cells/mL.^44^

For 200 ns pulse treatment [200 ns, 100 pulses, 10 Hz, ΔV (Table 1)] and 100 μs pulse treatment [100 μs, 8 pulses, 1 Hz, ΔV (Table 1)], 150 μL was transferred into 2 mm aluminum cuvettes (VWR International, USA), while in the case of 4 ns pulse treatment [4 ns, Δ number of pulses, 0.5 kHz, 12 kV] the volume was limited (due to impedance matching between generator and biological load) to 60 μL. Pulse generators, current/voltage probes, and oscilloscopes described in our recent study^41^ were the same experimental setup used. Voltage in Table 1 is based on voltage settings on pulse generator. Delivered voltage was approximately 50% lower in case of 200 ns pulses, while 100 μs are delivered consistent with generator settings.^41^

**Table 1.** Voltages (V) of Experimental Points for 200 ns and 100 µs Pulse Treatment and Pulse Number (*n*) of Experimental Points for 4 ns and 100 µs Pulse Treatment for MMP Analysis in CHO and H9c2 Cell Line.

	CHO			H9c2		
	4 ns (*n*)	200 ns (V)	100 µs (V)	4 ns (*n*)	200 ns (V)	100 µs (V)
Sham control	0	0	0	0	0	0
20 % permeability	500	1000	100	250	1000	100
50 % permeability	1000	1250	150	500	1500	150
90 % survival	1500	2000	200	1000	2000	200
50 % survival	3000	3000	350	2000	2750	350
20 % survival	4000	4000	475	4000	4000	500

Differences in voltages/pulse number reflect different electroporation intensities.

For permeability assay, cells were mixed with Propidium Iodide (PI, Life Technologies) to a final concentration of 100 μg/mL before pulse treatment. Three minutes after treatment, the sample was removed from the cuvette and the uptake of PI in cells was analyzed by the flow cytometer (Attune NxT; Life Technologies, Carlsbad, CA, USA) using 488 nm blue laser and 574/26 nm band-pass filter. The analysis of 10.000 events was performed by Attune NxT software. On the dot-plots of forward-scatter and side-scatter, the debris and clusters were excluded from the analysis. Fluorescence intensity histograms were used to determine the percentage of PI-permeabilized cells. Gating was set according to sham control (0V). Measurements for each data point were repeated three times.

In viability assay, immediately after electroporation, fresh growth media were added to the samples to obtain 5 x10^5^ cell/mL. Then 80 μL of diluted sample was transferred to a 96-well plate (TPP, Switzerland) and incubated at 37°C and humidified in 5% CO_2_ atmosphere for 24 h. According to the manufacturer’s instructions (CellTiter 96 AQueous One Solution Cell Proliferation Assay, Promega, USA), 20 μL of MTS tetrazolium compound was added to the samples, and after 2 h the absorbance of formazan (reduced MTS tetrazolium compound) was measured with a spectrofluorometer (Tecan Infinite M200, Tecan, Austria) at 490 nm. A percentage of viable cells was obtained by the normalization of sample absorbance to the absorbance of the control (0V). Each sample was performed in three technical repetitions and measurements for each sample were repeated three times.

**FIG. 1.**
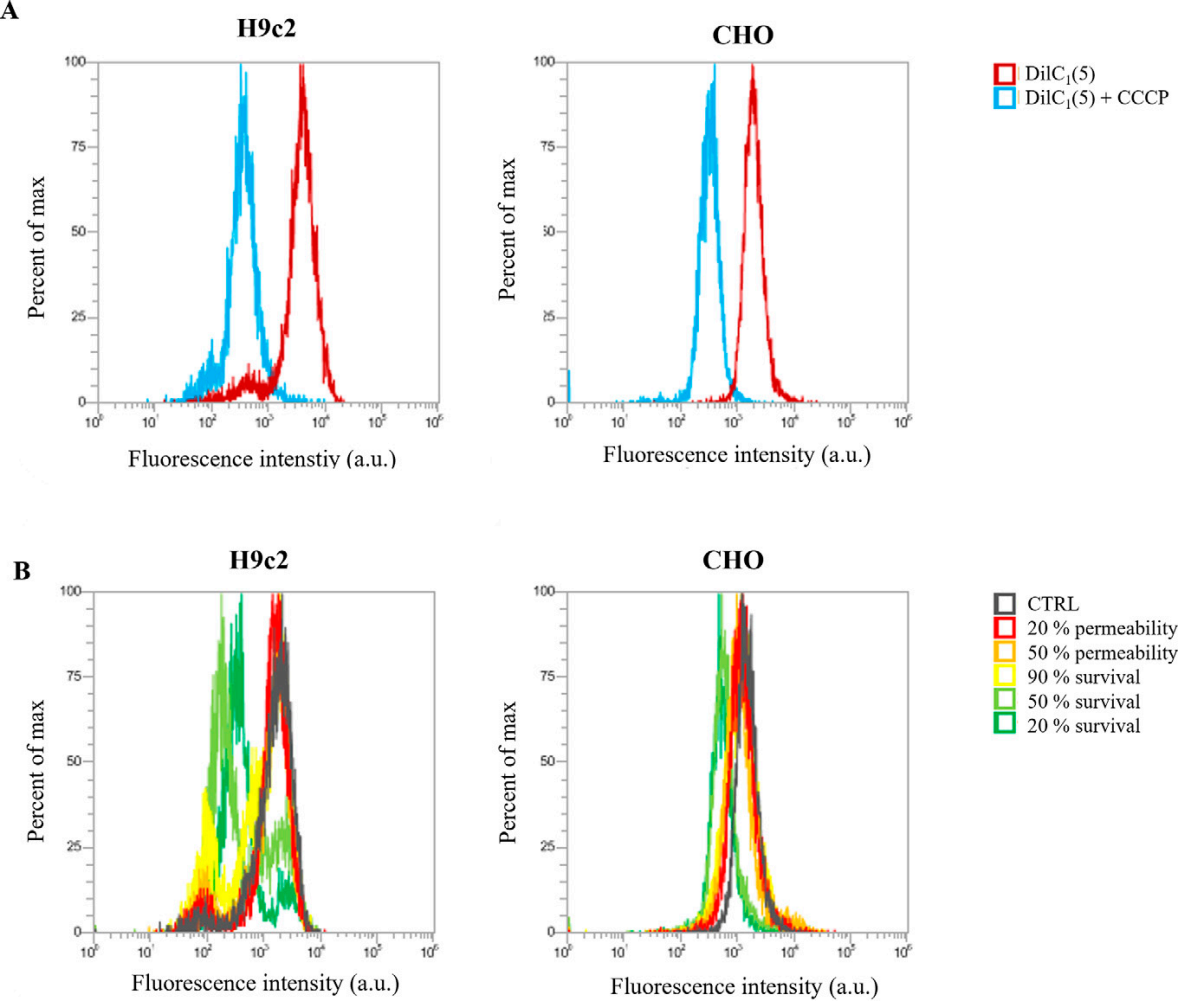
**(A)** MMP signal in control (red line) and CCCP-treated sample (blue line). **(B)** Changes in MMP signal after different electroporation intensities in 4 ns pulse treatment, detected 30 min after pulse treatment. MMP, mitochondrial membrane potential; CCCP, carbonyl cyanide m-chlorophenyl hydrazone; ns, nanosecond.

**FIG. 2.**
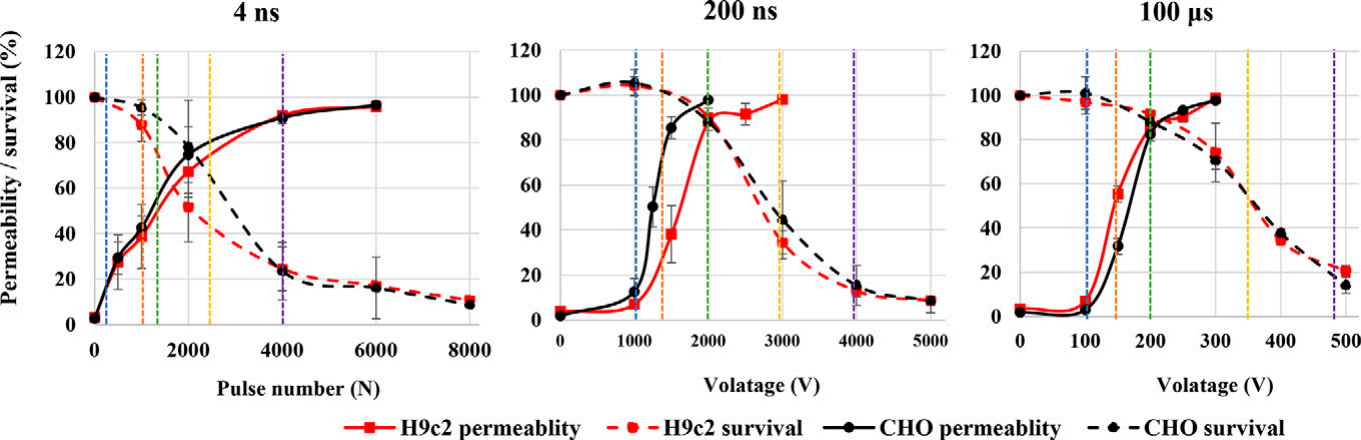
Permeability and survival curves on H9c2 (red) and CHO (black) cell line. Blue line shows approximate voltage needed for electroporation intensity leading to 20% permeability, orange line for 50% permeability, green line for 90% survival, yellow line for 50% survival, and violet line for 20% survival.

**FIG. 6.**
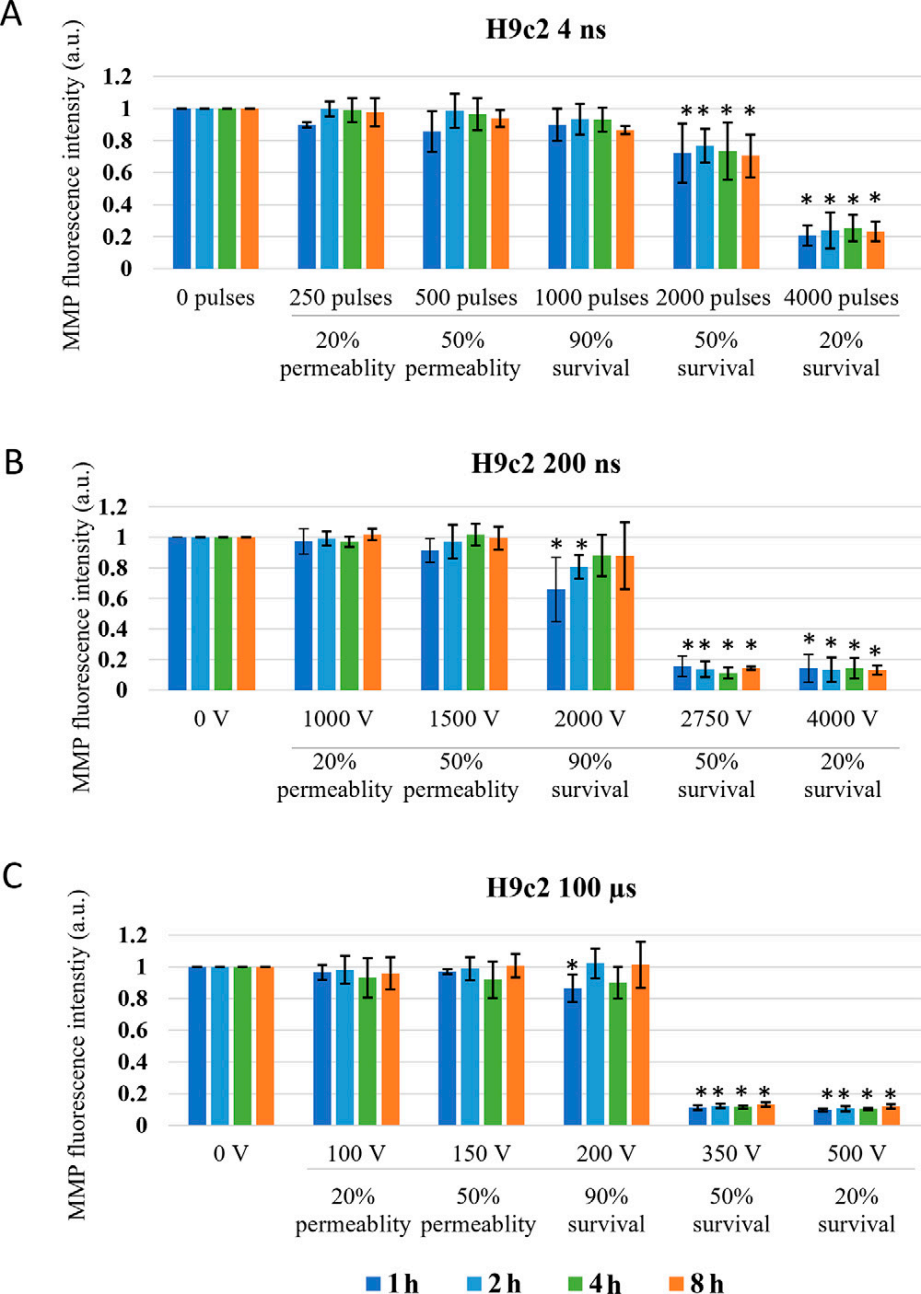
Changes in MMP in H9c2 cells exposed to different electroporation intensities at 1 (dark blue), 2 (light blue) and 4 (green) and 8 (orange) h after pulse treatment are presented as mean with standard deviation (±). Asterisk (*) above column show statistically significant changes (*p* < 0.05) between electroporation intensity and sham control within each time point. Statistically significant changes (*p* < 0.05) between time points in one electroporation intensity are present with connecting line. **(A)** 4 ns pulses, **(B)** 200 ns pulses, and **(C)** 100 µs pulses.

Mitochondrial membrane potential assay: Experimental points for MMP were determined on permeability and survival curves (Fig. 2). Experimental points are presented in Table 1. MMP was detected with the MitoProbe™ DiIC1(5) Assay Kit (M34151, Invitrogen™, Thermo Fisher, USA). DiIC1(5) penetrates the cytosol of eukaryotic cells and accumulates primarily in mitochondria with active MMP, producing bright, far-red fluorescence. DiIC1(5) staining intensity decreases with decreased MMP. Carbonyl cyanide m-chlorophenyl hydrazone (CCCP) is a MMP disrupter, supplemented in the assay kit, which was used for positive control. Changes in MMP were detected with flow cytometer (Attune NxT) using 637 nm red laser and 670/14 nm band-pass filter. The analysis of 10.000 events was performed by the Attune NxT software. On the dot-plots of forward-scatter and side-scatter, the debris and clusters were excluded from the analysis. Fluorescence intensity histograms were used to determine the changes in MMP signal (Fig. 6). Data on median fluorescence of MMP signal for each sample were collected. Measurements for each data point were repeated three times. Within every time point, result was normalized to the sham control, including positive CCCP control. MMP changes after 4 ns pulse treatment 30 min after pulse treatment are presented in Figure 1, however, the trend is similar in one cell line with all pulses at all time points.

Due to the vast time scale, two modes of the assay were used. For MMP detection, minutes after pulse treatment, cells were stained with DiIC1(5) before pulse treatment. Around 1 mL of cells was transferred to 1.5-mL tube mixed with DiIC1(5) (7 μL for H9c2 and 2 μL for CHO) and incubated in the incubator for 20 min. Afterward, 150 μL of stained cell suspension (60 μL for 4 ns) was transferred to 2 mm cuvettes and pulses were delivered. Immediately after electroporation, every sample was transferred to a new 1.5-mL tube through the plastic pipette. Samples for immediate detection of MMP changes were analyzed by flow immediately (1 min after pulse treatment), while samples for 30- and 60-min analyses were returned to the incubator until the analysis time. These samples were lightly shaken every 15 min to avoid attachment of the cells. When time for analysis was reached, samples’ MMP signal was detected on flow. While 150 μL was sufficient for flow analysis, 60 μL was not, therefore additional 60 μL of media were added before flow analysis.

For MMP investigation at later time points (1, 2, 4, and 8 h after pulse treatment), 150 μL of cell suspension (60 μL for 4 ns) was transferred to 2 mm cuvettes and pulses were delivered. Immediately after pulse delivery, samples were diluted in fresh media and 1.5 × 10^4^ cells were plated in a 24-well plate with 1 mL of growth medium. Cells were then returned to the incubator until analysis. For analysis cells, were harvested (unattached and attached) with trypsinization, transferred to 1.5-mL tubes, and centrifuged at 500 *g* for 1 min. Meanwhile, dye was prepared. DiIC1(5) (7 μL for H9c2 and 2 μL for CHO) was added to 1 mL of fresh medium. Cell pellet was resuspended in 150 μL of dye suspension and incubated at 37°C for 20 min. Afterward, samples were analyzed on flow. For MMP-positive control, 4 μL of stock solution CCCP was added to the DiIC1(5) dyed cells, incubated for 5 min in the incubator, and analyzed on flow.

Statistical analysis was performed using SigmaPlot 11.0 (Systat Software, USA). Statistically significant differences (*p < 0.05) were determined by one-way ANOVA test and Holm–Sidak *post hoc* test.

## Results

Based on permeability and survival curves, electroporation intensities leading to 20% and 50% permeability and 90%, 50%, and 20% survival were chosen (Fig. 2, Table 1).

**FIG. 3.**
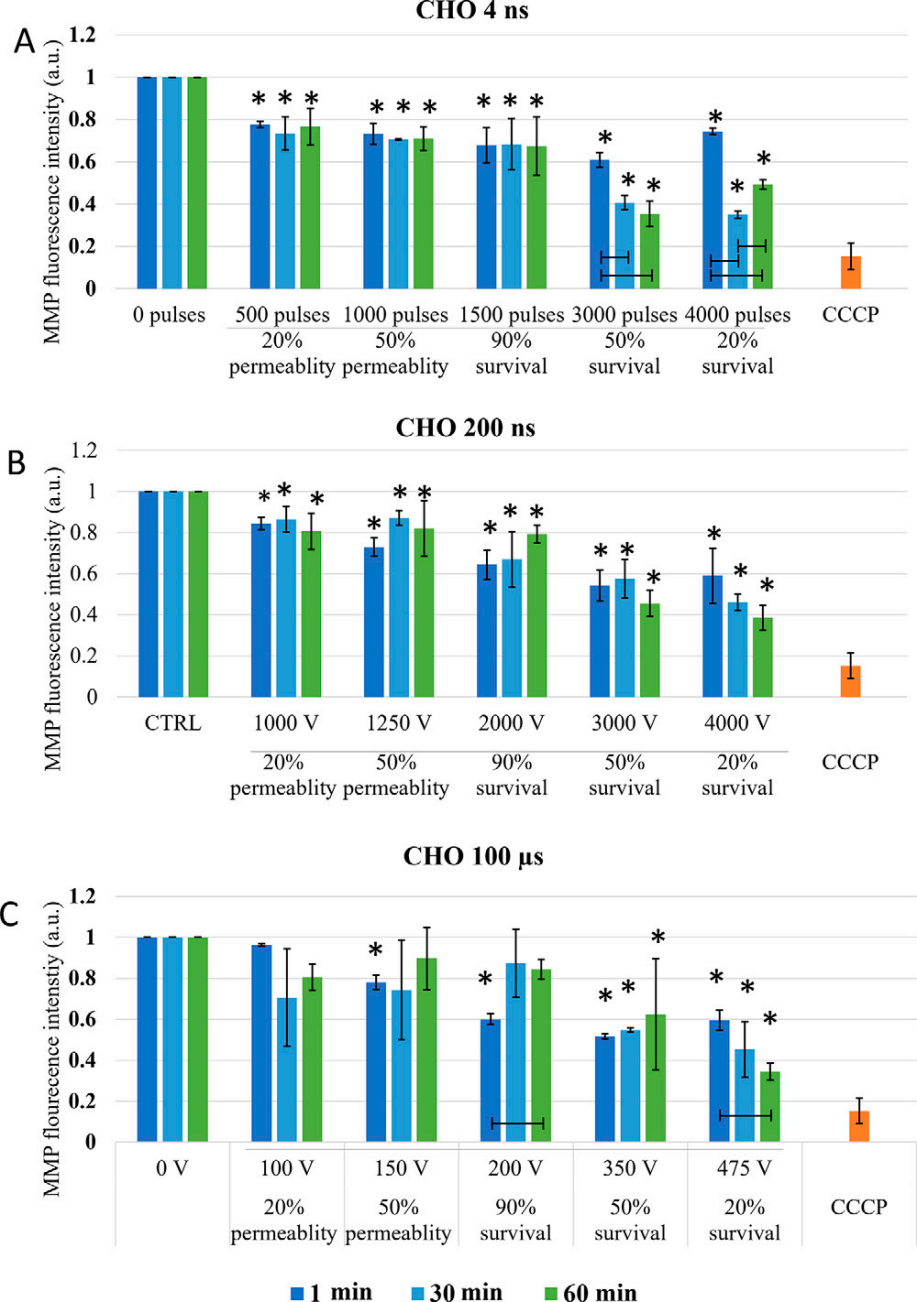
Changes in MMP in CHO cells exposed to different electroporation intensities at 1 (dark blue), 30 (light blue), and 60 (green) min after pulse treatment are presented as mean with standard deviation (±). Asterisk (*) above column show statistically significant changes (*p* < 0.05) between electroporation intensity and sham control within each timepoint. Statistically significant changes (*p* < 0.05) between time points in one electroporation intensity are present with connecting line. CCCP-positive control is presented in orange. **(A)** 4 ns pulses, **(B)** 200 ns pulses, and **(C)** 100 µs pulses.

Changes in MMP were investigated at the specific pulse amplitudes at different time points after electroporation to observe dynamics of MMP changes, i.e., immediate changes (changes in MMP within one hour) and delayed changes (changes up to 8 h) in two cell lines. In both CHO and H9c2 cell line, MMP was decreasing with the increase in electroporation intensity. Threshold for MMP decrease was different in two cell lines used in the study. In CHO (Fig. 3) 4 and 200 ns pulses decrease MMP immediately (1 min after pulse treatment) already at the lowest electroporation intensity, i.e., where 20% permeability (as determined by PI) was detected. With 100 μs pulses, immediate (1 min after pulse treatment) MMP decrease was detected at 50% permeability. This is a higher electroporation intensity compared with ns pulses, however, still well within the range of reversible electroporation. After ns treatment, changes in MMP detected immediately after pulse treatment persisted also at 30 and 60 min, while in μs pulse treatment MMP increased at 30 and 60 min after pulse treatment, suggesting activation of some mitochondrial membrane repair mechanism. This trend was observed at 90% survival, i.e., also 90% permeability, but not at higher amplitudes.

With the increase of electroporation intensity (resulting in increase of cell death-50 and 20% survival), MMP decreased even further in CHO cells (Fig. 3). This was observed in all pulse treatments, regardless of pulse duration. MMP signal between 1, 30, and 60 min, even though not statistically significant with 200 ns, MPP decreased further with time, showing also dynamic changes in MMP at high electroporation intensity, which results in cell death.

**FIG. 4.**
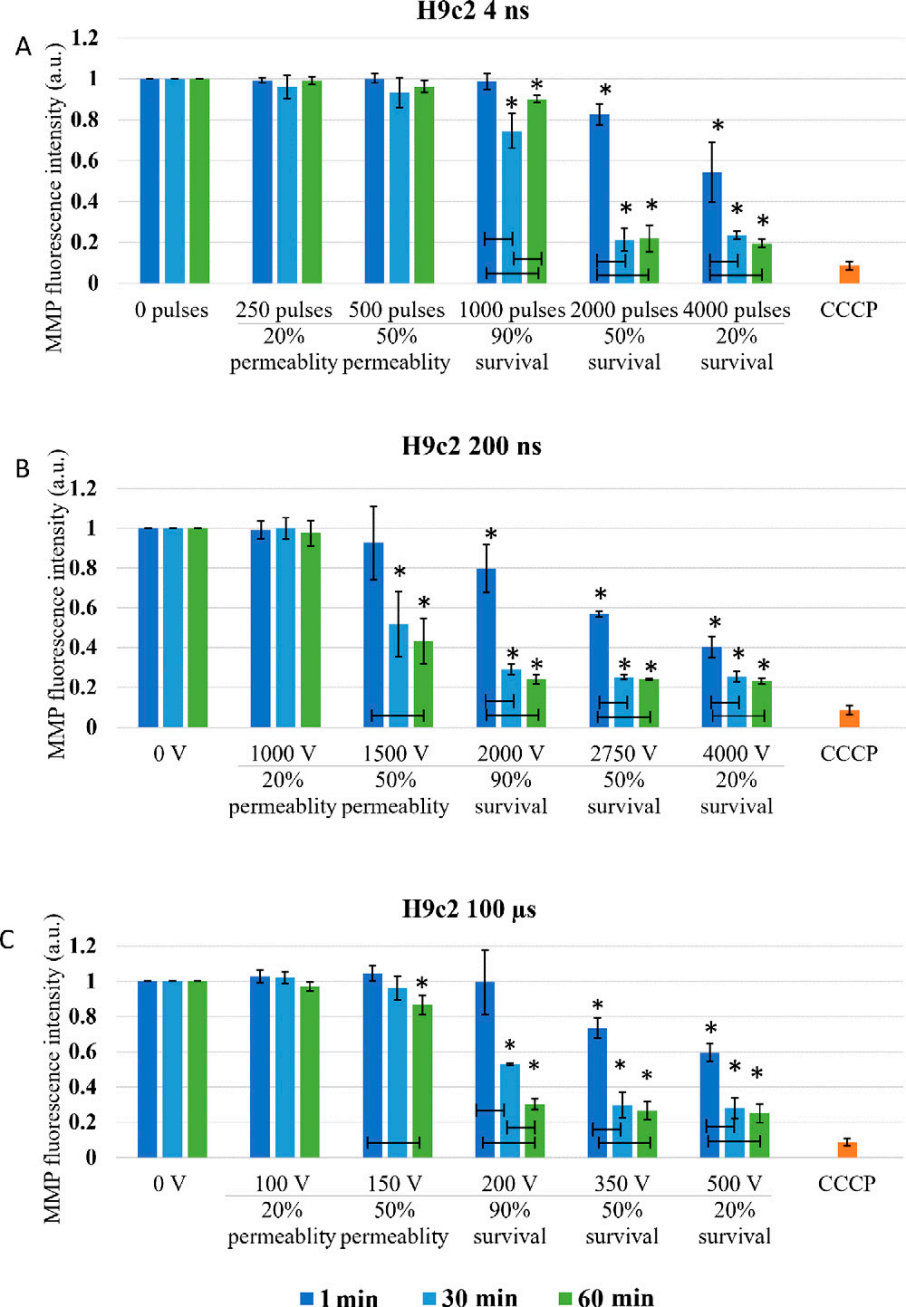
Changes in MMP in H9c2 cells exposed to different electroporation intensities at 1 (dark blue), 30 (light blue), and 60 (green) min after pulse treatment are presented as mean with standard deviation (±). Asterisk (*) above column show statistically significant changes (*p* < 0.05) between electroporation intensity and sham control within each time point. Statistically significant changes (*p* < 0.05) between time points in one electroporation intensity are present with connecting line. CCCP-positive control is presented in orange. **(A)** 4 ns pulses, **(B)** 200 ns pulses, and **(C)** 100 µs pulses.

The threshold for immediate changes in MMP (1 min after pulse treatment) in H9c2 (Fig. 4) was observed at pulse treatment leading to 50% permeability. In contrast to CHO cell line in H9c2 cells, μs pulses showed the same efficiency in MMP decrease as 200 ns pulses, while 4 ns pulses needed higher electroporation intensity. Delayed decrease (30 and 60 min after pulse treatment) in MMP was observed with all pulse treatments.

With the increase of electroporation intensity leading to decrease in survival (50 and 20% survival) MMP decreased even further. This decrease is clearly dynamic as in all pulse treatments, MMP signal decreases even further with prolonged time analysis (from 1 to 60 min).

**FIG. 5.**
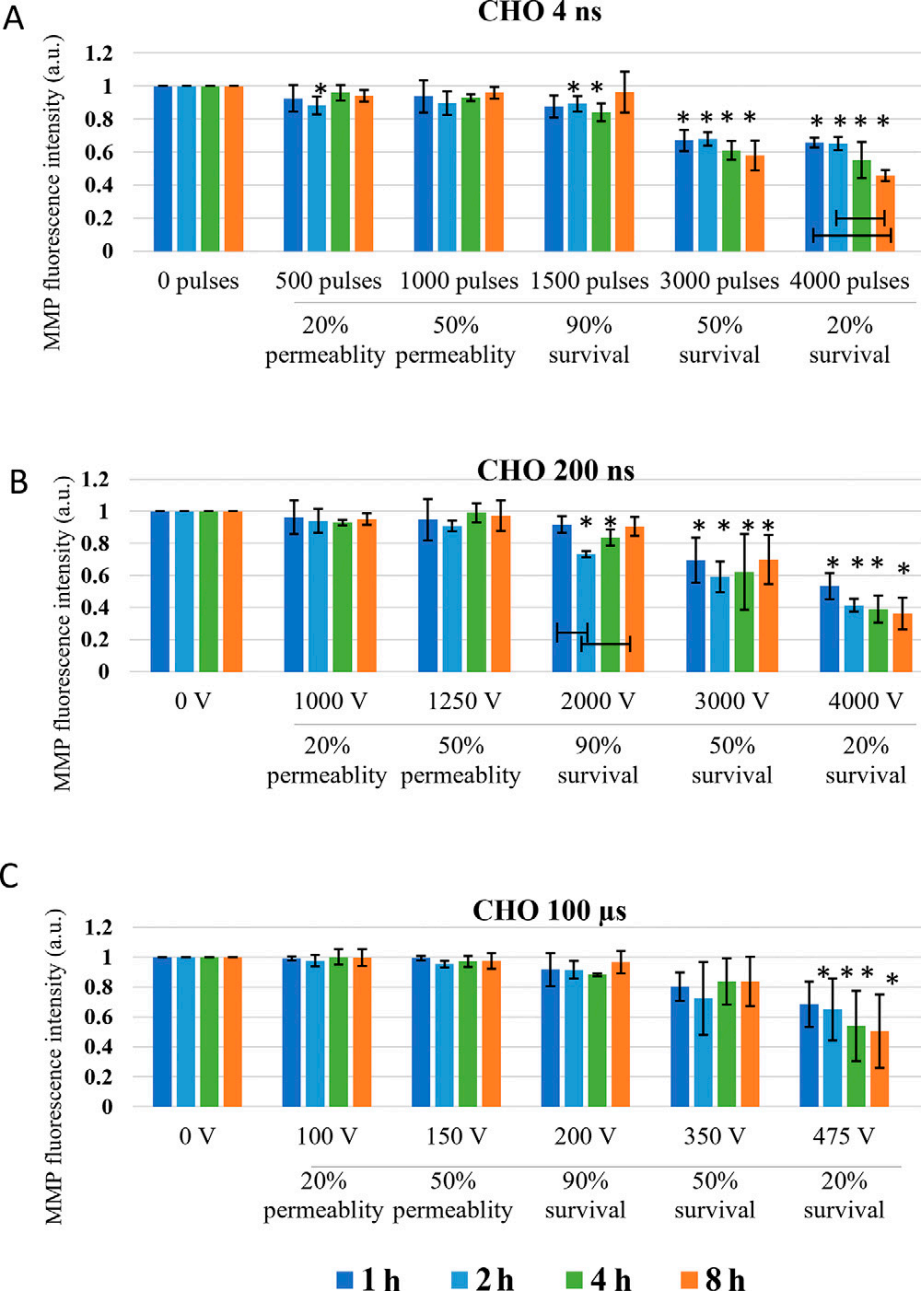
Changes in MMP in CHO cells exposed to different electroporation intensities at 1 (dark blue), 2 (light blue) and 4 (green) and 8 (orange) h after pulse treatment are presented as mean with standard deviation (±). Asterisk (*) above column show statistically significant changes (*p* < 0.05) between electroporation intensity and sham control within each time point. Statistically significant changes (*p* < 0.05) between time points in one electroporation intensity are present with connecting line. **(A)** 4 ns pulses, **(B)** 200 ns pulses, and **(C)** 100 µs pulses.

Long-lasting effect of pulse treatment on MMP changes was investigated at 1, 2, 4, and 8 h after treatment. In CHO (Fig. 5), no decrease in MMP signal in the range of reversible electroporation was detected (except for 4 ns pulse treatment at 2 h). In the range of irreversible electroporation, decrease in MMP was detected in all pulse treatments. Few statistically significant dynamical changes in MMP were detected between 1 and 8 h after pulse treatment, however, in general, dynamical changes in MMP were not detected.

In H9c2 (Fig. 6), decrease in MMP was detected at electroporation intensity leading to 90% survival for 200 ns and 100 μs and at electroporation intensity leading to 50% survival for 4 ns pulses. At electroporation intensity leading to 90% survival, MMP changes were detected in the first hour after electroporation and were no longer detected at 4 or 8 h after treatment. However, at electroporation intensity leading to 50 and 20% survival, decrease in MMP was present at all investigated time points. This decrease was much more evident compared with CHO cell line. Furthermore, no statistically significant dynamical changes in MMP were detected between 1 and 8 h after pulse treatment in all electroporation intensities.

## Discussion

Comparison of pulse treatments with different pulse durations is difficult, due to other parameters like repetition frequency, pulse number, and electric field needed to achieve permeabilization and different cell death pathways. Therefore, we compared them on the level of efficacy on plasma membrane permeability and viability, i.e., electroporation intensity. Investigation of changes in MMP at different intensities of electroporation can provide information how pulses are affecting mitochondrial membrane before viability is affected, i.e., in the range of reversible electroporation and when cells are dying, i.e., in the range of irreversible electroporation.

Based on the theory that ns pulses cause permeabilization of mitochondrial membrane directly,^5,23^ (which longer pulses presuming do not), decrease in MMP was expected in both CHO and H9c2 cell lines after application of 4 and 200 ns, while MMP in 100 μs treatment should be unaffected. According to our result, this theory fails. According to MMP analysis performed immediately (1 min) after pulse treatment in CHO cells, both 4 and 200 ns pulse cause decrease already in 20% permeability (Fig. 3). Similar decrease in MMP was detected also with 100 μs pulses at 50% permeability i.e., at 100% viability. In H9c2, 200 ns pulses and 100 μs pulses, had the same efficacy in decreasing MMP (at 50% permeability), while 4 ns pulses needed even higher electroporation intensity (Fig. 4). Our results indicate that longer pulses decrease MMP just like ns pulses: immediately after pulse treatment and already in the range of reversible electroporation, i.e., where cell viability is not yet affected.

Together with additional analysis performed at 30 and 60 min, dynamics of MMP decrease was analyzed. In CHO cells, MMP dynamics were observed only with 4 ns treatment, while in H9c2 all treatments (in most cases) resulted in even lower MMP, which indicates that changes in MMP are dynamic within 1 hour after pulse treatment. Different dynamic profiles of MMP changes to the same pulse treatment demonstrate different cell response, indicating cell type dependency. Interestingly ns pulse treatment was reported to cause different cell response in different cell lines or even within one cell line (benign, malignant, or aggressive form of cancer).^23,45^

In the range of irreversible electroporation, MMP decreases even further regardless of the pulse duration and cell type (Figs. 3 and 4). Like in reversible electroporation, a decrease in the range of irreversible electroporation was already observed after 1 min, suggesting a direct effect on mitochondrial membrane. However, MMP decreased even lower with time, indicating that indirect effects on mitochondrial membrane are (also) involved.

Looking at hours after pulse treatment, we observed repair of mitochondrial membrane and reestablishing MMP, as changes in MMP were no longer detected in the range of reversible electroporation (Figs. 5 and 6). Interestingly, MMP changes detected 60 min after pulse treatment in CHO and H9c2 in the range of reversible electroporation, were no longer detected in the subsequent analysis after 1–8 h. The reason for this is probably in different staining step of MMP assay, as once MMP dye DiIC_1_(5) was added before pulse treatment, and the second time after pulse treatment. In the first case, all cells were stained since all cells were alive and had normal functional mitochondria with high MMP and the results show direct effect on mitochondria. In the second case, staining is performed hours after electroporation and also after trypsinization. In hours after electroporation during the incubation, dead cells may degrade,^40^ leading to the analysis to incomplete cells’ sample, which could limit our data interpretation. The rate of this degradation may even explain the different values of MMP signal in the range of irreversible electroporation between CHO and H9c2 cell line. Also, H9c2 as muscle cells contain more mitochondria than CHO cells, making the changes in mitochondria more pronounced. In the range of irreversible electroporation, decrease in MMP was observed with both ns and μs treatments. MMP decreased with the increase of electroporation intensely. The decrease in MMP was in CHO and H9c2 sometimes observed already with electroporation intensely leading to 90%, but was consistently observed with electroporation intensely leading to 50% and 20% survival. At these conditions, the observed decrease in MMP is probably related to increase in cell death, i.e., population with abolishing mitochondrial energy production, as shown previously with metabolic MTS assay, where reduction of MTS tetrazolium compound is used to measure the mitochondrial metabolic rate, which indirectly reflects cell viability.^40^ In addition, it seems that dynamic changes can only be detected minutes after electroporation, as there were no changes observed in MMP detected at 1, 2, 4, and 8 h after treatment (Figs. 5 and 6). For 4 ns and 100 μs pulses, this agrees also with our previous study where no changes in MTS-assessed viability/metabolic rate were detected between 1, 2, 4, and 8 h after treatment in both H9c2 and CHO cell lines, regardless of the electroporation intensity. However, 200 ns pulse treatment causes some dynamic changes in MTS-assessed viability/metabolic rate with electroporation intensities affecting survival in both cell lines, however, these changes are more pronounced in CHO cell line.^40^

Detection of changes in MMP signal has previously been used in multiple electroporation studies, but only with ns pulse treatment. However, it seems that general reagents for detecting changes in mitochondrial potential may not be the most appropriate of electroporation studies, or at least their use is limited. While problems with marking mitochondria for MMP after electroporation are explained, there should also be some considerations in interpretation of changes in MMP due to electroporation. From changes in MMP, we can indirectly assume the changes on the mitochondrial membrane—in case of electroporation treatment on the permeability of the mitochondrial membrane. However, these changes in MMP can also be explained by proton (H^+^) changes. MMP is generated by proton pumps of respiratory chain, leading to high concentration of H^+^ in intramembrane space. These H^+^ are then used by the ATPase as the driving force to bind adenosine diphosphate (ADP) and Pi and release ATP. The levels of MMP and ATP in the cell are kept relatively stable and sustained changes in MMP or ATP may be deleterious.^24^ Electroporation causes leakage of ATP to extracellular space already, e.g., ATP intracellular ATP depletion, in the range of reversible electroporation immediately after pulse treatment.^46–48^ Electroporation, as cell membrane injury, also triggers repair mechanism, which require additional ATP, thus further boosting ATP production.^49^ This ATP must be replaced for the cell to survive, leading to the increased activity of ATPases. Increased ATP production would lead to higher proton use and thus reduce the proton gradient across the inner mitochondrial membrane, therefore, lowering the MMP. Furthermore, if electroporation pulses directly affect mitochondrial membrane, proteins in mitochondrial membrane can also be affected in a similar way to proteins in plasma membrane during plasma membrane electroporation.^1^ Such protein could be ATPases, and its deformation could open a pathway for protons, which would result in lower MMP. For sure there are also other possible explanations why MMP is decreased even in the absence of creating electroporation pores in mitochondrial membrane. Therefore, changes in MMP in electroporation studies should be interpreted with caution.

## Conclusion

Previous reports claim that an increase with ns treatment in pulse number or electric field leads to lower MMP signal. Our results are consistent with published data as higher intensity of ns pulse treatment results in lower MMP signal, presumably due to changes in mitochondrial membrane permeability. Yet within this study, we have shown that also 100 μs pulses cause similar changes in MMP in both reversible and irreversible electroporation range. It seems that all pulses affect plasma membrane and MMP in a similar way, making intracellular membrane electroporating properties of ns pulses questionable and suggesting that MMP is potentially affected through secondary pathway as a result of plasma membrane permeabilization due to electroporation. In addition, response of CHO in H9c2 to pulse treatment were somewhat different suggesting some differences could also be cell type dependent.
